# Proteomics-Based Approach for Detailing the Allergenic Profile of Cannabis Chemotypes

**DOI:** 10.3390/ijms241813964

**Published:** 2023-09-11

**Authors:** Cali Loblundo, Jenna Severa, Gabrielle A. Peruggia, Kristina Reid Black, Suman Chandra, Hemant Lata, Mahmoud ElSohly, Martin D. Chapman, Deepak A. Deshpande, Ajay P. Nayak

**Affiliations:** 1 Department of Medicine, Center for Translational Medicine, Thomas Jefferson University, Philadelphia, PA 19107, USA; cali.loblundo@students.jefferson.edu (C.L.); jenna.severa@students.jefferson.edu (J.S.); gabrielleperuggia@gmail.com (G.A.P.); deepak.deshpande@jefferson.edu (D.A.D.); 2 InBio, Charlottesville, VA 22903, USA; kreidblack@inbio.com (K.R.B.); mdc@inbio.com (M.D.C.); 3 National Center for Natural Product Research, Research Institute of Pharmaceutical Sciences, School of Pharmacy, University of Mississippi, Oxford, MS 38677, USA; suman@olemiss.edu (S.C.); hlata@olemiss.edu (H.L.); melsohly@olemiss.edu (M.E.); 4 Division of Pulmonary, Allergy and Critical Care Medicine, Jane and Leonard Korman Respiratory Institute, Thomas Jefferson University, Philadelphia, PA 19107, USA

**Keywords:** cannabis, allergens, proteomics, diagnostics

## Abstract

Allergic sensitization to cannabis is an emerging public health concern and is difficult to clinically establish owing to lack of standardized diagnostic approaches. Attempts to develop diagnostic tools were largely hampered by the Schedule I restrictions on cannabis, which limited accessibility for research. Recently, however, hemp was removed from the classified list, and increased accessibility to hemp allows for the evaluation of its practical clinical value for allergy diagnosis. We hypothesized that the proteomic profile is preserved across different cannabis chemotypes and that hemp would be an ideal source of plant material for clinical testing. Using a proteomics-based approach, we examined whether distinct varieties of cannabis plant contain relevant allergens of cannabis. Cannabis extracts were generated from high tetrahydrocannabinol variety (Mx), high cannabidiol variety (V1-19) and mixed profile variety (B5) using a Plant Total Protein Extraction Kit. Hemp extracts were generated using other standardized methods. Protein samples were subjected to nanoscale tandem mass spectrometry. Acquired peptides sequences were examined against the Cannabis sativa database to establish protein identity. Non-specific lipid transfer protein (Can s 3) level was measured using a recently developed ELISA 2.0 assay. Proteomic analysis identified 49 distinct potential allergens in protein extracts from all chemotypes. Most importantly, clinically relevant and validated allergens, such as profilin (Can s 2), Can s 3 and Bet v 1-domain-containing protein 10 (Can s 5), were identified in all chemotypes at label-free quantification (LFP) intensities > 106. However, the oxygen evolving enhancer protein 2 (Can s 4) was not detected in any of the protein samples. Similarly, Can s 2, Can s 3 and Can s 5 peptides were also detected in hemp protein extracts. The validation of these findings using the ELISA 2.0 assay indicated that hemp extract contains 30–37 ng of Can s 3 allergen per µg of total protein. Our proteomic studies indicate that relevant cannabis allergens are consistently expressed across distinct cannabis chemotypes. Further, hemp may serve as an ideal practical substitute for clinical testing, since it expresses most allergens relevant to cannabis sensitization, including the validated major allergen Can s 3.

## 1. Introduction

In recent years, cannabis has become increasingly accessible for medicinal and recreational use owing to the evolving social perception and the legalization efforts in many states across US. Concomitantly, there has also been an increase in reports of allergic sensitization to cannabis [1]. Further, a large workforce participates in various operations within the cannabis industry, leading to concerns of allergic sensitization in occupational environments [2,3,4,5]. More recently, one case of mortality was reported in a licensed cannabis growing facility with occupational asthma identified as the probable cause (ref: Occupational Safety and Health Administration, Inspection #1572011). Although cannabis allergy has emerged as a public health issue, its prevalence is unclear due to broad symptomology and a lack of diagnostic approaches to clinically establish a link between sensitization and disease [1,6]. Nevertheless, studies in our laboratory and elsewhere have identified and subsequently validated multiple allergens, including profilin (Can s 2), non-specific lipid transfer protein (nsLTP; Can s 3), oxygen evolving enhancer protein 2 (OEEP2; Can s 4) and Bet v 1-domain-containing protein (Can s 5) [3,7,8,9,10,11]. Initially, Can s 3 was reported as a major allergen of Cannabis in Europe but not in North America [3]; however, more recently, our laboratory established Can s 3 as a relevant allergen in North America [12]. Taken together, IgE immunoreactivity to specific allergens partly explains allergic sensitization to cannabis, indicating a possible role for other allergens.

However, investigations into additional allergens and the development of diagnostic tests is somewhat hindered by practical considerations. Firstly, although broadly divided into indica and sativa cultivars, over 11,000 distinct strains of Cannabis exist, mostly arising from the crossing of different cannabis varieties, resulting in hybrid strains selected for unique biochemical profiles. Thus, accounting for the distinct allergenic profile of each variety is not feasible. Consequently, it becomes essential to develop a practical approach to separate varieties on the basis of other features, such as a biochemical profile. Classifying cannabis plants by chemotypes aligns with real-world use scenarios where cannabis use is generally dependent on the primary purpose of use (i.e., mood-altering effects, anti-inflammatory effects, anti-emetic effects). More specifically, cannabis varieties are defined by cannabinoid quality, which is the ratio of THC and CBD, and by genetic characteristics [13], thus providing a practicable system for comparing allergenic profiles between different cannabis varieties. While cannabis continues to be classified as a Schedule I substance, protein extracts generated from any variety of cannabis (including strains > 0.3% THC) are exempt from restrictions. Further, the impact of Schedule I limitations has been further mitigated through the 2018 Farm Bill, which legalized hemp production for various purposes. Therefore, we questioned (1) whether the distinct chemotypes of cannabis express recently validated allergens and (2) whether hemp is a suitable alternative for testing allergic sensitization to hemp.

In the present study, we used the tandem mass spectrometry (MS/MS) approach to resolve the proteomic profile of four distinct chemotypes of cannabis, including a high tetrahydrocannabinol (THC) variety (Mx), a high cannabidiol (CBD) variety (V1-19), a mixed profile variety (B5) and hemp. Proteomics analysis indicates that relevant allergens of cannabis are expressed in all strains of cannabis, including hemp. Further, Can s 3, a major allergen of cannabis, is expressed at high levels in hemp, suggesting clinical utility in diagnostics.

## 2. Results

### 2.1. Nanoscale LC-MS/MS Analysis

**Figure 1:**
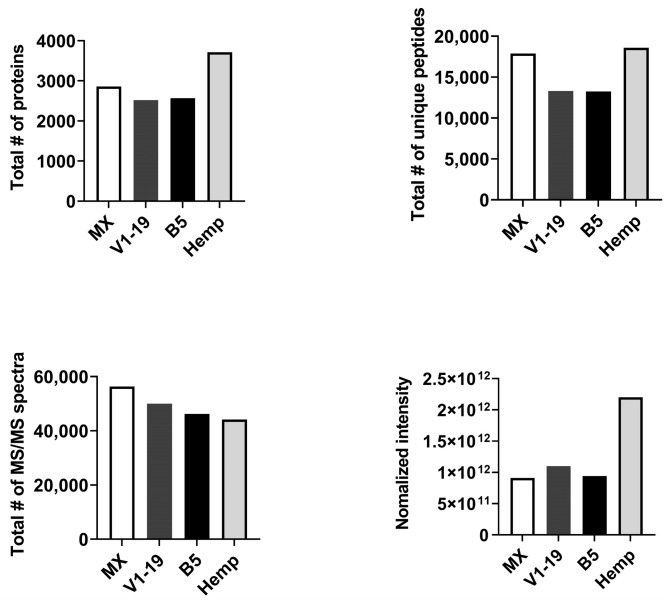
Overview of MS/MS analysis of distinct cannabis chemotypes and hemp. Total number of proteins identified (**top left panel**), total number of unique peptides recovered (**top right panel**), total number of tandem mass spectra (MS/MS) (**bottom left panel**) and total normalized label-free quantification (LFQ) intensity (**bottom right panel**).

Approximately 2500–3000 total number of proteins were identified in protein extract generated from the three distinct cannabis chemotypes, while hemp ~3700 proteins were identified in the protein extract generated from hemp (Figure 1, top left panel). Further, the total number of unique peptides identified in each sample was >13,000, being highest for hemp (18,592), followed by the Mx (17,889), V1-19 (13,310) and B5 (13,237) chemotypes of cannabis (Figure 1, top right panel). The total number of MS/MS spectra acquired were comparable between all strains of cannabis, with the Mx strain yielding maximum MS/MS spectra (Figure 1, bottom left panel). Finally, regarding the normalized LFQ intensity, while comparable between the three distinct cannabis chemotypes (range 9.1 × 10^11^–1.1 × 10^12^), LFPQ intensity was highest for the hemp strain (2.2 × 10^12^) (Figure 1 bottom right panel). It is essential to note here that while quantitative measures can be compared between the Mx, V1-19 and B5 strains of cannabis, hemp extracts were generated using distinct protein extraction method and analyzed separately, thus yielding differential LFQ intensities that limit direct quantitative comparisons.

### 2.2. Putative Allergens of Cannabis

**Figure 2:**
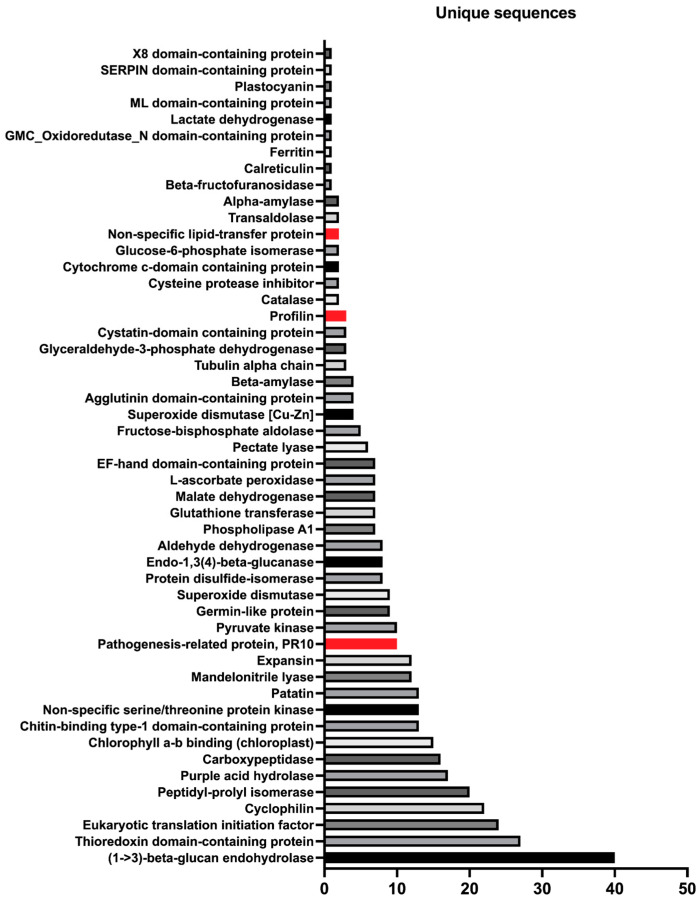
Putative allergens in cannabis detected via the LC-MS/MS approach. Graph represents the number of unique sequences identified for each putative allergen. Validated allergens from cannabis are highlighted in red.

**Figure 3:**
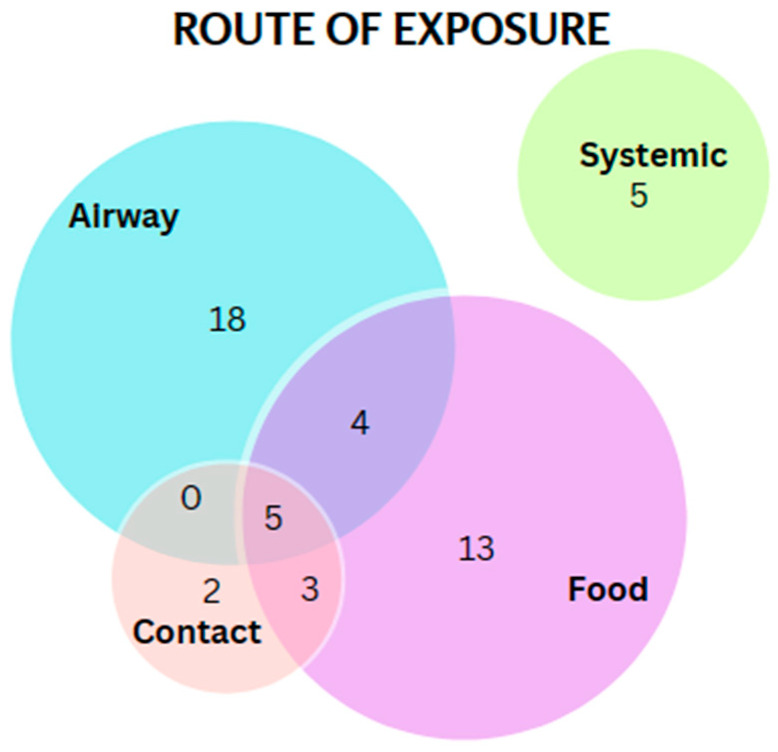
Distribution of putative allergens of cannabis by route of exposure.

Preliminary analysis revealed the presence of cannabis proteins belonging to 50 distinct allergen families (Figure 2). Importantly, we identified peptides belonging to known and validated allergens of cannabis, including profilin (Can s 2), nonspecific lipid transfer protein (nsLTP, Can s 3) and Bet v 1-homologue- and pathogenesis-related protein PR-10 (Can s 5). Interestingly, we did not identify peptides for the oxygen-evolving enhancer protein 2 (OEEP2; Can s 4)) in cannabis extract samples in all chemotypes and hemp. Since allergic sensitization to cannabis can occur through multiple routes (inhalation, contact, ingestion), we examined the broad distribution of putative allergens based on the route of exposure (Figure 3 and Supplementary Table S1). The two putative contact allergens include proteins homologous to glucose–methanol–choline (GMC) oxidoreductase N-domain-containing protein (Mala s 12; skin yeast) and malate dehydrogenase (Mala f 4).

### 2.3. Putative Airway Allergens of Cannabis

**Figure 4:**
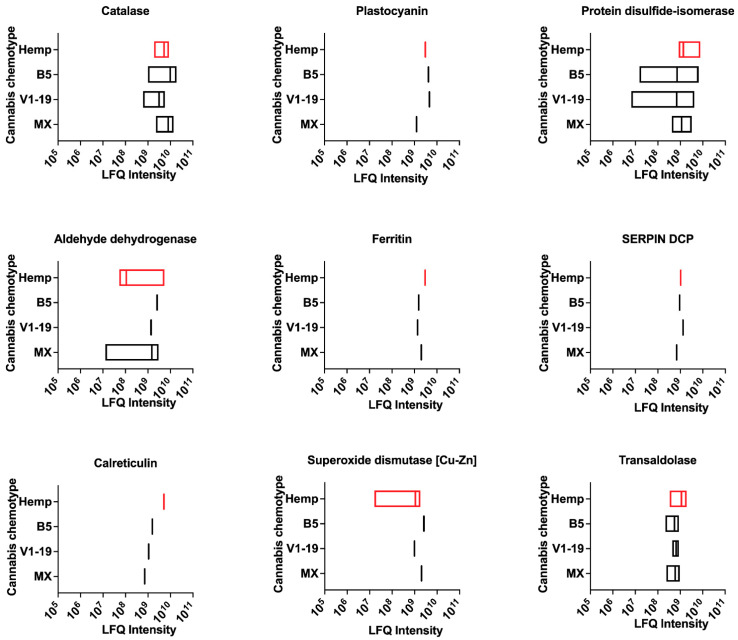

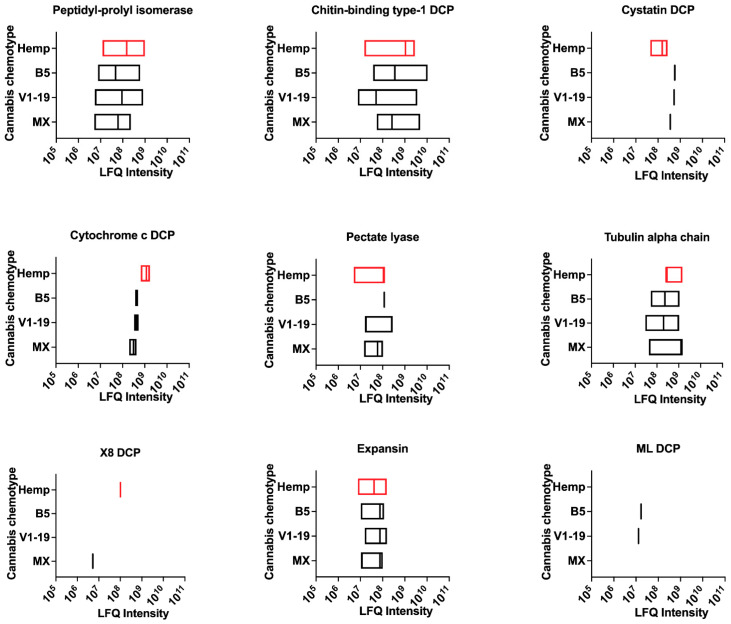
Putative airway allergens in cannabis chemotypes and hemp. LFQ intensities for putative airway allergens present in cannabis. DCP—domain-containing protein. LFQ—relative label-free quantification. Data variance represents LFQ intensity for different unique sequences in the database for each protein.

The primary route of consumption of cannabis continues to be the inhalation of cannabis smoke. Further, in symptomatic exposure to cannabis, one major concern is the exacerbation of underlying asthma. Consequently, allergens that are known to be sensitizers in airways are likely to be important. We identified 18 putative cannabis allergens in our LC-M/MS analysis that were previously reported as typical airway allergens (Figure 4 and Supplementary Table S1). Among these are allergens that share homology with common airway allergen sources, such as Dermatophagoides pteronyssius or D. farinae (house dust mite) (tubulin α-chain, Der p 33; ferritin, Der p 30; SERPIN domain-containing protein, Der f 27; Chitin-binding type-1 domain-containing protein, Der p 18). Some airway allergens in cannabis share homology with other mites, such as ML domain-containing protein and aldehyde dehydrogenase (Blo t 2 and Tyr p 35; storage mites), or grass allergens, such as expansin (Cyn d 1; Bermuda grass), pectate lyase (Amb a 1; short ragweed) and plastocyanin (Amb a 7; short ragweed). Other allergens include those with homology to fungal allergens, such as catalase (Pen c 30; Penicillium), protein disulfide-isomerase (Alt a 4; Alternaria), aldehyde dehydrogenase (Alt a 10; Alternaria), calreticulin (Pen ch 31; Penicillium), transaldolase (Cla c 14; Cladosporium), peptidyl-prolyl isomerase (Asp f 11; Aspergillus), cytochrome c-domain-containing protein (Cur l 3; Curvularia). Collectively, our data indicate that cannabis contains allergens that share significant homology with known allergens from other environmental sources. Further, peptides for putative cannabis allergens relevant to airway exposure were detected in all chemotypes of cannabis and in hemp. The two exceptions included the X8 domain-containing protein (hemp and Mx varieties) and ML-domain-containing protein (B5 and V1-19 varieties).

### 2.4. Putative Food Allergens of Cannabis

**Figure 5:**
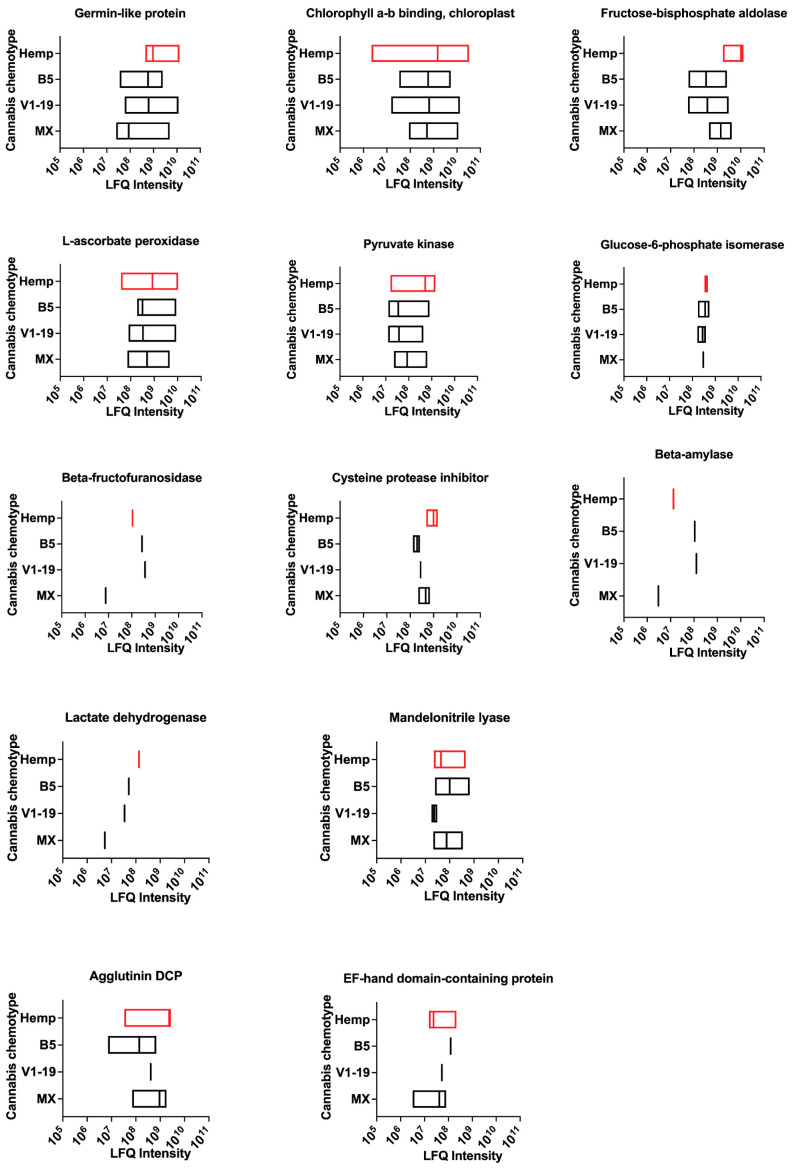
Putative food allergens in cannabis chemotypes and hemp. LFQ intensities for putative food allergens present in cannabis. DCP—domain-containing protein. LFQ—relative label-free quantification. Data variance represents LFQ intensity for different unique sequences in the database for each protein.

The ingestion of cannabis is common and is typically consumed in the form of tea or an ingredient in baked goods, and allergic reactions (including anaphylaxis) on the ingestion of cannabis has been reported in the literature [14,15]. We identified peptides for 13 putative food allergens in cannabis (Figure 5 and Supplementary Table S1), which include Germin-like protein (Cit s 1; sweet orange), Chlorophyll a-b-binding, chloroplast (Api g 3; celery), L-ascorbate peroxidase (Mus a 6; banana), β-fructofuranosidase (Sol a 12; tomato), cysteine protease inhibitor (Sola t 3; potato), β-amylase (Tri a 17; wheat), (R)-mandelonitrile lyase (Pru du 10; almond) and agglutinin domain-containing protein Tri a 18; wheat) peptides. Other putative food allergens shared homology with common striped catfish allergens, such as fructose-bisphosphate aldolase (Pan h 3), pyruvate kinase (Pan h 9), glucose-6-phosphate isomerase (Pan h 11) and lactate dehydrogenase (Pan h 10). Peptides for all putative food allergens in cannabis were detected in all chemotypes and hemp.

### 2.5. Putative Contact Allergens of Cannabis

**Figure 6:**
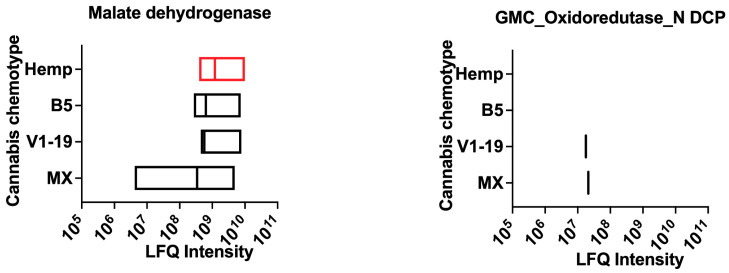
Putative contact allergens in cannabis chemotypes and hemp. LFQ intensities for putative contact allergens present in cannabis. DCP—domain-containing protein. LFQ—relative label-free quantification. Data variance represents LFQ intensity for different unique sequences in the database for each protein.

Contact exposure to cannabis can occur in both recreational and occupational settings. In LC-MS/MS analysis we identified two putative allergens in cannabis with homology to known contact allergens identified in skin yeasts (Figure 6 and Supplementary Table S1). These include malate dehydrogenase (Mala f 4) and GMC oxidoreductase N-domain-containing protein (Mala s 12). While malate dehydrogenase peptides were identified in all chemotypes of cannabis and hemp, peptides for GMC oxidoreductase N-domain containing protein were detected only in Mx and V1-19 varieties.

### 2.6. Putative Allergens of Cannabis with Capability to Sensitize through Multiple Routes

**Figure 7:**
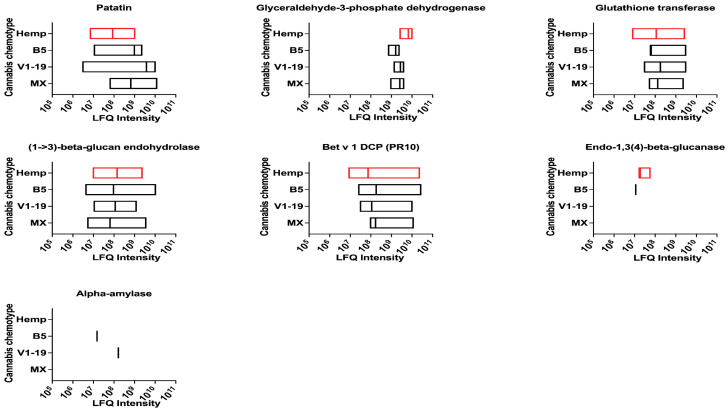
Dual allergens in cannabis chemotypes and hemp. LFQ intensities for seven potential dual routes (allergen–food or contact–food) allergens in cannabis. DCP—domain-containing protein. LFQ—relative label-free quantification. Data variance represents LFQ intensity for different unique sequences in the database for each protein.

Some allergens have been previously reported to cause allergic reactions through multiple routes. Most putative allergens share homology to common and potent allergens from birch (Betula verrucosa), latex (Hevea brasiliensis) and peanut (Arachis hypogea). Four allergens have been previously known to act as airway and food allergens concomitantly and include glyceraldehyde 3-phosphate dehydrogenase (Pan h 13 and Tri a 34), glutathione transferase (Der p 8, Asc l 13), α-amylase (Der p 4, Hor v 16) and Bet v 1-domain-containing protein (pathogenesis-related protein, PR-10) (Bet v 1, Ara h 8) (Figure 7 and Supplementary Table S1). Some allergens have been previously reported as sensitizers through contact and oral route and include endo-1,3(4)-beta-glucanase (Hev b 2, Mus a 5) and patatin (Sola t 1, Hev b 7). While peptides for α-amylase were detected only in the B5 and V1-19 varieties of cannabis, peptides for endo-1,3(4)-beta-glucanase were detected in hemp and B5 varieties.

**Figure 8:**
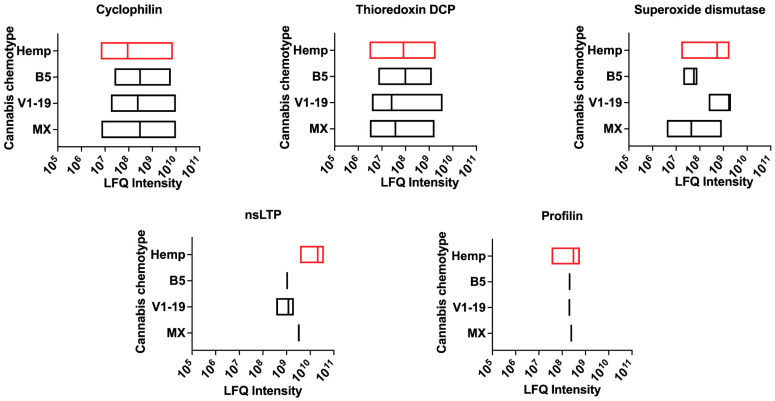
Multiple route allergens in cannabis and hemp. LFQ intensities for five potential multiple routes (allergen–food–contact) allergens in cannabis. DCP—domain-containing protein. LFQ—relative label-free quantification. Data variance represents LFQ intensity for different unique sequences in the database for each protein.

Five other allergens have been reported as sensitizers through all three routes and include cyclophilin (peptidyl-prolyl cis-trans isomerase) (Ara h 18, Bet v 17 and Mala s 6), thioredoxin domain-containing protein (Asp f 28, Mala s 13 and Tri a 15), superoxide dismutase (Asp f 6, Hev b 10, Pis v 4). The validated allergens of cannabis, including nonspecific lipid transfer protein (Can s 3) (with homology to Hev b 12, Amb a 6, Pru p 3) and profilin (Can s 2) (with homology to Bet v 2, Hev b 8 and Ara h 5) were also detected and are known to sensitize via multiple routes (Figure 8 and Supplementary Table S1).

### 2.7. Additional Validation of Cannabis Allergens

**Figure 9:**
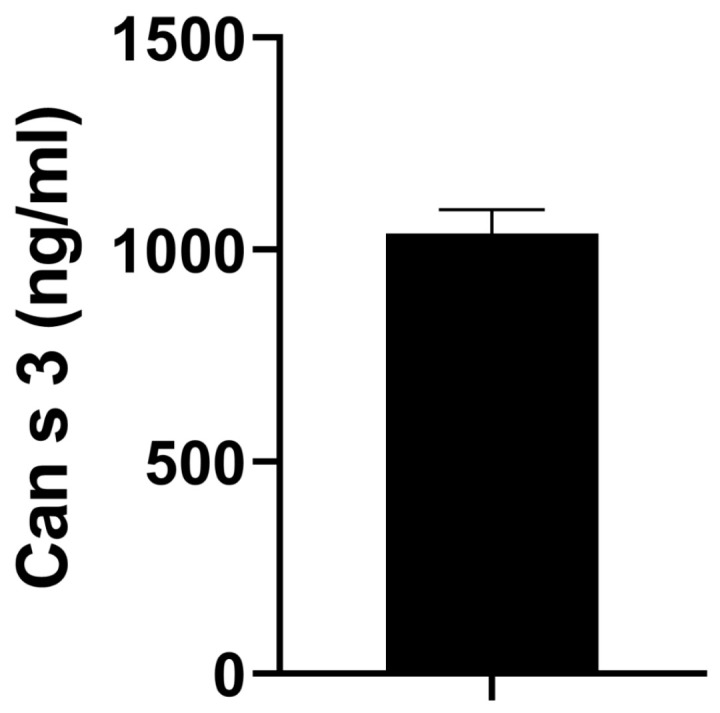
ELISA assay. ELISA assay measuring Can s 3 levels in hemp protein extract samples. Hemp protein lysates consistently reported ~35 µg/mL of Can s 3 allergen.

Our LC-MS/MS analysis revealed the consistent expression of validated allergens of cannabis in all chemotypes of cannabis and hemp tested in this study. Interestingly, the major cannabis allergen nonspecific lipid transfer protein or Can s 3 evidently had one of the highest LFQ intensities among all proteins detected in hemp. To determine the exact concentration of Can s 3 allergen in hemp extracts, we used an ELISA assay [16] and were able to measure ~1 μg of Can s 3 allergen in hemp samples, which translated to ~35 ng of Can s 3 normalized to 1 μg of total hemp protein extract (Figure 9).

## 3. Discussion

Our unbiased quantitative proteomics approach revealed the presence of at least 50 different proteins in cannabis that share homology with known allergens. Our analysis also revealed that most proteins are consistently expressed in the distinct chemotypes of cannabis. Most importantly, almost all allergens are expressed in hemp and the major allergen of cannabis, Can s 3 (nonspecific lipid transfer protein or nsLTP), is highly expressed in hemp. We were able to detect ~35 ng of Can s 3 per μg of hemp protein extract. Collectively, our studies underscore the potential for hemp as a suitable representative to in clinical diagnostics to establish type I hypersensitivity to cannabis proteins.

Our preliminary analysis of peptide acquisition and protein sequences from LC-MS/MS revealed higher counts for hemp. Further, the total LFQ intensity was also very high for hemp extracts. This could be possibly explained by the method used for generating protein extracts compared to the method used for isolating proteins from the three different chemotypes of cannabis (Mx, B5 and V1-19 strains).

While we detected almost 50 putative allergens in cannabis, we do not suggest that all have relevance to allergic sensitization to cannabis. Eventually, the route of exposure to cannabis may determine the relevance of one allergen over another. Allergens with the ability to sensitize through multiple routes are likely to be important for further studies. Indeed, Can s 2 (profilin), Can s 3 (nsLTP) and Can s 5 (Bet v 1-homolog) are all important allergens of cannabis, and proteins belonging to these allergen families can sensitize through more than one route. All three validated allergens were consistently detected in all chemotypes and hemp extracts. However, we did not detect peptides for the fourth validated allergen from cannabis, the oxygen evolving enhancer protein 2 (OEEP2 or Can s 4), across all strains tested in this study. One possibility is that OEEP2 might degrade under reducing conditions that were used for generating protein extracts.

Five other putative cannabis allergens have been associated with exposure through insect bites, and these include homologues of Api m 3 (purple acid hydrolase; honeybee), Api m 9 (carboxypeptidase; honeybee), For t 1, (nonspecific serine/threonine protein kinase; biting midge), For t 2 (eukaryotic translation initiation factor 3; biting midge) and Ves m 1 (phospholipase A1; yellowjacket). It is highly unlikely that putative allergens in cannabis variants that share homology with these proteins are relevant to sensitization. However, reports have emerged that cannabis injected via an intravenous route [17] could lead to anaphylaxis.

One limitation of our study is the direct quantitative correlation of proteins identified in hemp with other cannabis chemotypes. While we can secure hemp for research use within our facility, accessibility to cannabis strains, particularly those containing >0.3% THC are subjected to Schedule I restrictions. Nevertheless, our proteomics approach reveals that relevant cannabis allergens are consistently expressed across distinct cannabis chemotypes. Moreover, the major allergen of cannabis, the nonspecific lipid transfer protein Can s 3, is expressed at high levels in hemp. Owing to the practical challenges of using cannabis strains with >0.3% THC, hemp may serve as an ideal practical substitute for clinical testing. More specifically, skin prick testing (a routine allergen sensitization testing) and allergen challenge (inhalational or oral) with standardized hemp protein extracts may be essential tools for determining allergic sensitization to cannabis in clinic.

## 4. Materials and Methods

### 4.1. Cannabis Strains

Cannabis sativa strains with distinct chemical profile were cultured at the National Center for Natural Products Research at University of Mississippi. Three strains, including a high tetrahydrocannabinol (THC) variety (Mx), a high cannabidiol (CBD) variety (V1-19) and a mixed profile variety (B5) were grown using standard practices [18,19]. Hemp strain (White CBG) was obtained from Groff North America Hemplex (Red Lion, PA, USA). The strain is rich in cannabigerolic acid (CBGa; ~10%) with 0.108% THC (<0.3% cutoff for hemp strains) and 0% CBD.

### 4.2. Protein Extraction

Protein extraction was performed using two different methods. For the 3 strains Mx, V1-19 and B5, proteins were extracted from buds at the National Center for Natural Product Research, Research Institute of Pharmaceutical Sciences, School of Pharmacy at the University of Mississippi, using the PierceTM Plant Total Protein Extraction Kit (Thermo Fischer Scientific, Waltham, MA, USA) as per methodologies described previously [3]. Hemp protein extract was generated from buds by finely grinding buds using a mortar and pestle. One gram of hemp bud extract was transferred into a 15 mL conical tube and protein extraction buffer was performed under denaturing conditions using proprietary methods developed by InBio Inc. (Charlottesville, VA, USA).

### 4.3. Protein Estimation

Total protein content in cannabis plant extracts was measured using the PierceTM bicinchoninic acid (BCA) Protein Assay kit (Thermo Fisher Scientific), as per the manufacturer’s instructions. The serial dilution of cannabis protein samples and protein standards (generated with bovine serum albumin, BSA) were plated onto a 96-well plate, mixed with BCA working reagent solution mix and incubated at 37 °C for 30 min. Following incubation, plates were cooled briefly at room temperature (RT) and colorimetric absorbance signal was measured at 560 nm using a spectrophotometer. A standard curve was generated using absorbance measures at 560 nm for BSA protein standards. Absorbance signals measured for cannabis protein extracts was plotted on the standard curve to estimate protein concentrations.

### 4.4. Sample Preparation and Nanoscale Tandem Mass Spectrometry (MS/MS)

Cannabis protein sample preparation for LC-MS/MS analysis was performed using methods described previously [20]. All methods related to LC-MS/MS were performed by MS Bioworks (Ann Arbor, MI). Briefly, protein samples were first precipitated in tricarboxylic acid (TCA). Pellets were resolubilized in buffer containing 8 M urea, 50 mM Tris HCl, 150 mM NaCl, pH 8.0 and 1X Roche Complete Protease Inhibitor. Protein quantitation was performed using Qubit assay (ThermoFisher Scientific) and samples were normalized to the protein yield. The sample was digested overnight with trypsin (enzyme–substrate ratio of 1:20) with gentle shaking. Next, samples were reduced for 1 h at RT in 12 mM dithiothreitol (DTT) followed by alkylation for 1 h at RT in 15 mM iodoacetamide. Each sample was acidified in formic acid and subjected to solid-phase extraction (SPE) on a Waters μHLB C18 plate.

A total of 2 μg aliquot of samples was analyzed using a nano LC-MS/MS with a Waters NanoAcquity HPLC system interfaced to a ThermoFisher Fusion Lumos. Peptides were loaded on a trapping column and eluted over a 75 μm analytical column at 350 nL/min; both columns were packed with Luna C18 resin (Phenomenex). A 4 h gradient was employed. The mass spectrometer was operated in data-dependent mode, with MS and MS/MS performed (3 s cycles) in the Orbitrap at 60,000 full width at half maximum (FWHM) resolution and 15,000 FWHM resolution, respectively, with advanced peak determination (APD) activated.

### 4.5. Data Processing and Analysis

Data were processed through the MaxQuant software v1.6.2.3 (www.maxquant.org accessed on 23 June 2021). MS acquired data were recalibrated, and the data were filtered at 1% protein and peptide false discovery rate (FDR). LFQ Intensity was calculated from the individual peptide peak areas (extracted ion chromatograms) that were scaled to a protein value and then normalized between samples based on the total intensity per sample [21]. Data were saved on the Andromeda platform [22] using following criteria: enzyme—trypsin; database—UniProt (Cannabis sativa); fixed modification—carbamidomethyl (C); variable modifications—oxidation (M) and acetyl (protein N-terminal); and fragment mass tolerance—20 ppm. MaxQuant settings were as follows: protein and peptide FDR—0.01 FDR; minimum peptide length—7; minimum razor + unique peptides—1; minimum unique peptides—0; minimum ratio count for LFQ—1; and secondary peptide setting—TRUE. This was to account for two peptides co-transmitted and producing a composite product ion spectrum. Data were uploaded to Perseus v1.5.5.3 for data processing and analysis.

### 4.6. Identification of Putative Allergens in Cannabis

The peptide scan on the UniProt Cannabis sativa database revealed accession number identifiers (IDs) for cannabis proteins. Cannabis sativa sequence database scans revealed protein sequences with multiple open reading frames (ORFs), resulting in multiple unique protein accession IDs for some proteins. The protein lists for each cannabis chemotype and hemp were examined individually in the World Health Organization and the International Union of Immunological Societies (WHO/IUIS) allergen database to identify cannabis proteins belonging to existing allergen families.

## Data Availability

The data presented in this study are available on request from the corresponding author.

## Supplementary Materials

The following supporting information can be downloaded at: https://www.mdpi.com/article/10.3390/ijms241813964/s1.
