# Supplementary material for: Proteomics-Based Approach for Detailing the Allergenic Profile of Cannabis Chemotypes
Source: Int J Mol Sci. 2023 Sep 11;24(18):13964. doi: 10.3390/ijms241813964 (PMC10531008; doi:10.3390/ijms241813964)
Supplement: Supplementary file 1 [file ijms-24-13964-s001.zip › ijms-2550877-supplementary.pdf]

Table S1. Unique peptides of putative allergens present in Cannabis plant varieties. *N.D.* - not detected.

Protein ID #	FASTA Protein name	LFQ Intensity (MX strain)	LFQ Intensity (V1-19 strain)	LFQ Intensity (B5 strain)	LFQ Intensity (Hemp)	Homologous allergen (Source)	Route of exposure
**A0A7J6G6Y5 A0A7J6HK93**	**Purple acid phosphatase**	0.26E+10	**1.43E+10**	0.38E+10	2.7E+09	*Api m 3* (Honeybee)	Systemic
**A0A7J6G4E2**		2.99E+09	**7.75E+09**	6.37E+09	4.15E+09		
**A0A7J6G5V2 A0A7J6DP71**		2.52E+08	4.97E+08	**6.74E+08**	2.5E+08		
**A0A7J6HKU1**		0.71E+08	1.26E+08	1.63E+08	**5.6E+08**		
**A0A7J6HM49**		2.90E+07	**8.73E+07**	3.08E+07	3.3E+07		
**A0A7J6HM22**		**7.60E+07**	3.91E+07	5.88E+07	3E+07		
**A0A7J6HKG5 A0A7J6G4D3**		0.97E+07	**2.18E+07**	1.52E+07	*N.D.*		
**A0A7J6FG55 A0A7J6FXQ0**		*N.D.*	2.06E+07	**2.47E+07**	*N.D.*		
**A0A7J6F0B8**		1.58E+07	1.37E+07	3.52E+07	**5.4E+07**		
**A0A7J6GRN0**		*N.D.*	**1.35E+07**	*N.D.*	*N.D.*		
**A0A7J6F9L6 A0A7J6FM87**		*N.D.*	*N.D.*	**1.59E+07**	*N.D.*		

**A0A7J6G664**		*N.D.*	*N.D.*	**6.67E+07**	*N.D.*		
**A0A7J6EZ30**	**Germin-like protein**	0.49E+10	1.18E+10	0.25E+10	**1.3E+10**	*Cit s 1* (Sweet orange)	Food
**A0A7J6HWG7**		0.79E+09	**5.25E+09**	0.93E+09	9.5E+08		
**A0A7J6EL31**<br>**A0A7J6EXR1**		0.52E+09	**1.13E+09**	1.05E+09	4.5E+08		
**A0A7J6HB19**		0.62E+08	**1.12E+08**	0.36E+08	*N.D.*		
**A0A7J6HCP0**		0.87E+08	0.59E+08	**2.48E+08**	*N.D.*		
**A0A7J6I2N2**		3.09E+07	**5.87E+07**	*N.D.*	*N.D.*		
**A0A7J6DNM0**<br>**A0A7J6DQN0**		2.54E+07	*N.D.*	9.82E+07	*N.D.*		
**A0A7J6I4D6**<br>**A0A7J6FUP8**<br>**A0A7J6EHQ3**<br>**A0A7J6EKL2**	**Patatin**	**1.29E+10**	1.08E+10	0.24E+10	1.1E+09	*Hev b 7* (Latex rubber)<br>*Sola t 1* (Potato)	Contact<br>Food
**A0A7J6GFY3**<br>**A0A7J6GG48**		0.06E+09	**3.92E+09**	1.30E+09	3.2E+07		
**A0A7J6GCU9**<br>**A0A7J6GD72**		1.16E+09	**3.62E+09**	0.65E+09	1.4E+08		
**A0A7J6FBN8**<br>**A0A7J6GLU6**<br>**A0A7J6H6S6**<br>**A0A7J6E5A3**		**1.54E+08**	*N.D.*	1.02E+07	*N.D.*		

**A0A7J6I2Z0**		*N.D.*	2.87E+06	*N.D.*	**6.65E+06**		
**A0A7J6GUI2 A0A7J6ENV8**	**Chlorophyll a-b binding, chloroplast**	1.15E+10	1.08E+10	0.54E+10	**3.3E+10**	*Api g 3* (Celery)	Food
**A0A7J6E9Z3**		0.26E+10	**1.34E+10**	0.46E+10	5.3E+09		
**A0A7J6FCQ7**		1.49E+09	1.56E+09	1.89E+09	**4.8E+09**		
**A0A7J6GN45 A0A7J6I6P7 A0A7J6I1W9**		4.28E+08	7.43E+08	7.92E+08	**1.9E+09**		
**A0A7J6GN20**		5.24E+08	**7.33E+08**	5.98E+08	**1.1E+09**		
**A0A7J6I6H8**		2.99E+08	5.64E+08	5.23E+08	**1.2E+09**		
**A0A7J6I1T4**		6.46E+08	4.83E+08	5.84E+08	**8E+09**		
**A0A7J6ICI2**		1.82E+08	2.92E+08	2.46E+08	**3.2E+08**		
**A0A7J6GSM8**		0.88E+08	1.44E+08	0.97E+08	**5.1E+08**		
**A0A7J6GTJ9**		*N.D.*	1.55E+07	3.40E+07	**1.6E+09**		
**A0A7J6GAG5**		*N.D.*	*N.D.*	*N.D.*	**1.4E+09**		
**A0A7J6HU96**		*N.D.*	*N.D.*	*N.D.*	**2.23E+06**		
**A0A7J6EG53 A0A7J6H8I4 A0A7J6FMU3 A0A7J6HHS7 A0A7J6G0I6 A0A7J6FM26**	**Cyclophilin (Peptidyl-prolyl cis-trans isomerase)**	**9.94E+09**	9.85E+09	5.98E+09	7.33E+09	*Ara h 18* (Peanut)	Food

**A0A7J6FM12<br>A0A7J6DLL0**		1.62E+09	**2.17E+09**	0.84E+09	1.98E+09	*Asp f 27* (Fungi-Ascomycete)<br>*Bet v 7* (White birch)<br>*Cat r 1* (Rosy periwinkle)<br>*Der f 29* (House dust mite)<br>*Der p 29* (House dust mite)<br>*Mala s 6* (Skin yeast)<br>*Ole e 15* (Olive)<br>*Psi c 2* (Fungi-Basidiomycete)<br>*Sola l 5* (Tomato)	Airway<br>Airway<br>Airway<br>Airway<br>Airway<br>Contact<br>Airway<br>Airway<br>Food
**A0A7J6GX90<br>A0A7J6HKI4<br>A0A7J6E511**		2.98E+08	6.83E+08	5.59E+08	**1.06E+09**		
**A0A7J6ICC5**		5.99E+08	6.66E+08	7.18E+08	**7.85E+08**		
**A0A7J6EHL2**		3.84E+08	**4.02E+08**	3.00E+08	3.08E+08		
**A0A7J6FIH3**		7.46E+07	8.12E+07	8.48E+07	**9E+07**		
**A0A7J6FMC9**		*N.D.*	4.61E+07	**5.16E+07**	*N.D.*		
**A0A7J6H982**		*N.D.*	3.82E+07	*N.D.*	6.60E+06		
**A0A7J6HET7**		1.35E+07	**3.14E+07**	2.75E+07	*N.D.*		
**A0A7J6F3F0<br>A0A7J6ECK7**		**4.83E+07**	1.77E+07	2.51E+07	2.1E+07		
**A0A7J6I7K3**		7.04E+06	*N.D.*	*N.D.*	**7.08E+07**		
**A0A7J6GVF7**		*N.D.*	*N.D.*	*N.D.*	**3.4E+07**		
**A0A7J6I9I7**		*N.D.*	*N.D.*	*N.D.*	**2E+07**		
**A0A7J6EZ77**	**Malate dehydrogenase**	4.85E+09	7.74E+09	7.42E+09	**9.92E+09**	*Mala f 4* (Skin yeast)	Contact
**A0A7J6E8J3<br>A0A7J6F4C2**		4.27E+09	3.76E+09	3.71E+09	**8.79E+09**		
**A0A7J6E1Y2**		2.06E+08	5.72E+08	**6.36E+08**	5.03E+08		

**A0A7J6GGJ8**		0.42E+07	5.39E+08	**5.93E+08**	4.07E+08		
**A0A7J6EH81**		3.41E+08	4.43E+08	2.72E+08	**1.90E+09**		
**A0A7J6FBS2**		*N.D.*	*N.D.*	*N.D.*	**3.95E+08**		
**A0A7J6F3P9**	**Catalase**	1.39E+10	0.57E+10	**1.88E+10**	8.59E+09	*Pen c 30* (Fungi-Ascomycete)	Airway
**A0A7J6HVQ1**		**2.28E+09**	0.61E+09	1.00E+09	1.89E+09		
**A0A7J6E3I7**	**Plastocyanin**	1.24E+09	**4.62E+09**	4.20E+09	3.05E+09	*Amb a 7* (Short ragweed)	Airway
**A0A7J6HQK8**<br>**A0A7J6FZ57**	**Glyceraldehyde-3-phosphate dehydrogenase**	4.28E+09	4.23E+09	2.54E+09	**1.08E+10**	*Pan h 13* (Striped catfish)<br>*Per a 13* (Cockroach)<br>*Tri a 34* (Wheat)	Food<br>Airway<br>Airway
**A0A7J6FL67**		0.87E+09	1.26E+09	0.67E+09	**2.44E+09**		
**A0A7J6GU43**<br>**A0A7J6EJG0**<br>**A0A7J6FHP7**	**Protein disulfide-isomerase**	3.20E+09	4.20E+09	6.46E+09	**7.68E+09**	*Alt a 4* (Fungi-Ascomycete)	Airway
**A0A7J6E6I1**		1.13E+09	0.75E+09	0.59E+09	**2.06E+09**		
**A0A7J6FGV5**		4.20E+08	6.32E+08	8.31E+08	**1.36E+09**		
**A0A7J6HAJ3**		*N.D.*	0.66E+07	1.52E+07	**1.36E+09**		
**A0A7J6HM83**<br>**A0A7J6FQT2**		*N.D.*	*N.D.*	*N.D.*	**8.32E+08**		

**A0A7J6F8T7**	**Thioredoxin domain-containing protein**	1.68E+09	**3.67E+09**	1.30E+09	1.88E+09	*Asp f 28* (Fungi-Ascomycete)<br>*Asp f 29* (Fungi-Ascomycete)<br>*Cop c 2* (Fungi-Basidiomycete)<br>*Fus c 2* (Fungi-Ascomycete)<br>*Mala s 13* (Skin yeast)<br>*Plo i 2* (Moth)<br>*Tri a 15* (Wheat)<br>*Zea m 25* (Maize)	Airway<br>Airway<br>Airway<br>Airway<br>Contact<br>Airway<br>Food<br>Airway
**A0A7J6G4W4**<br>**A0A7J6EM56**		5.59E+08	4.91E+08	3.32E+08	**1.14E+09**		
**A0A7J6HKK7**<br>**A0A7J6GEH8**		0.82E+08	3.46E+08	1.37E+08	**4.01E+08**		
**A0A7J6DWJ5**		1.02E+08	2.40E+08	1.64E+08	**2.81E+08**		
**A0A7J6EP85**<br>**A0A7J6EBB3**		1.41E+08	**2.22E+08**	1.31E+08	1.34E+08		
**A0A7J6GEK2**		1.34E+08	**2.11E+08**	2.06E+08	1.41E+08		
**A0A7J6FRK6**<br>**A0A7J6GSW1**		3.30E+07	2.85E+07	**6.54E+07**	1.55E+07		
**A0A7J6HB22**<br>**A0A7J6DM82**		*N.D.*	2.27E+07	1.39E+07	**2.54E+07**		
**A0A7J6I4E0**		1.08E+07	**1.80E+07**	1.29E+07			
**A0A7J6H1C4**		1.02E+07	1.68E+07	0.70E+07	**2.08E+07**		
**A0A7J6FPC0**		0.31E+07	**1.37E+07**	1.03E+07			
**A0A7J6I370**		1.15E+07	1.01E+07	*N.D.*	**1.30E+08**		
**A0A7J6DN91**		*N.D.*	**7.2E+06**	*N.D.*			
**A0A7J6FL75**		1.62E+07	0.38E+07	1.08E+07	**9.25E+07**		
**A0A7J6GJ28**		**3.71E+07**	*N.D.*	*N.D.*			
**A0A7J6GHT3**		*N.D.*	*N.D.*	*N.D.*	**8.10E+07**		
**A0A7J6HAM6**		*N.D.*	*N.D.*	*N.D.*	**3.22E+07**		
**A0A7J6DS35**		*N.D.*	*N.D.*	*N.D.*	**2.18E+07**		
**A0A7J6E9X5**<br>**A0A7J6FRN3**		*N.D.*	*N.D.*	*N.D.*	**1.43E+07**		
**A0A7J6F172**		*N.D.*	*N.D.*	*N.D.*	**1.24E+07**		
**A0A7J6HIT6**		*N.D.*	*N.D.*	*N.D.*	**2.96E+06**		

**A0A7J6GR52**	**Glutathione transferase**	2.47E+09	**3.29E+09**	3.27E+09	2.84E+09	*Alt a 13* (Fungi-Ascomycete)<br>*Asc l 13* (Roundworm)<br>*Asc l 13* (Pig roundworm)<br>*Bet v 8* (White birch)<br>*Bla g 5* (Cockroach)<br>*Blo t 8* (Storage mite)<br>*Der f 8* (House dust mite)<br>*Der p 8* (House dust mite)<br>*Per a 5* (Cockroach)<br>*Tyr p 8* (Storage mite)	Airway<br>Food<br>Food<br>Airway<br>Airway<br>Airway<br>Airway<br>Airway<br>Airway<br>Airway
**A0A7J6F7P6**		1.79E+08	**3.18E+08**	*N.D.*	1.95E+08		
**A0A7J6F7M2**		1.70E+08	**2.47E+08**	0.64E+08	2.96E+07		
**A0A7J6I7R8**		0.96E+08	1.08E+08	**1.25E+08**	1.19E+07		
**A0A7J6F7K5 A0A7J6GPS7**		6.62E+07	6.31E+07	6.36E+07	**2.84E+09**		
**A0A7J6I7S2**		4.81E+07	2.77E+07	**5.42E+07**	7.41E+06		
**A0A7J6E5J2**	**Fructose-bisphosphate aldolase**	4.12E+09	3.02E+09	2.56E+09	**1.31E+10**	*Gad m 3* (Atlantic cod)<br>*Pan h 3* (Striped catfish)<br>*Sal s 3* (Atlantic salmon)<br>*Thu a 3* (Yellowfin tuna)	Food<br>Food<br>Food<br>Food
**A0A7J6EBM3**		1.40E+09	0.55E+08	0.58E+08	**1.30E+10**		
**A0A7J6GPM5**		4.38E+08	3.72E+08	3.27E+08	**1.73E+09**		

**A0A7J6FZU7<br>A0A7J6GZL8**		*N.D.*	*N.D.*	*N.D.*	**7.42E+09**		
**A0A7J6FS68<br>A0A7J6E4J8**	**Superoxide dismutase**	0.44E+08	**2.07E+09**	0.56E+08	6.36E+08	*Alt a 14* (Fungi-Ascomycete)<br>*Amb t 13* (Giant ragweed)<br>*Art si 8* (Wormwood)<br>*Asp f 6* (Fungi-Ascomycete)<br>*Hev b 10* (Latex rubber)<br>*Mala s 11* (Skin yeast)<br>*Pis v 4* (Pistachio)	Airway<br>Airway<br>Airway<br>Airway<br>Contact<br>Contact<br>Food
**A0A7J6FXY1<br>A0A7J6GEA8**		0.85E+09	**1.71E+09**	0.80E+08	4.45E+08		
**A0A7J6EWT4**		0.04E+08	**2.37E+08**	0.20E+08	4.15E+07		
**A0A7J6G1D4**		*N.D.*	*N.D.*	*N.D.*	**1.79E+09**		
**A0A7J6ELP6<br>A0A7J6HB40**		*N.D.*	*N.D.*	*N.D.*	**1.08E+09**		
**A0A7J6FFH3**		*N.D.*	*N.D.*	*N.D.*	**1.64E+07**		
**A0A7J6HF70**	**Non-specific lipid-transfer protein**	3.31E+09	2.02E+09	1.06E+09	**3.80E+10**	*Amb a 6* (Short ragweed)<br>*Ara h 9* (Peanut)<br>*Ara h 17* (Peanut)<br>*Cor a 8* (Hazelnut)	Airway<br>Food<br>Food<br>Food<br>Contact

**A0A7J6HF68**		*N.D.*	3.66E+08	*N.D.*	**3.70E+09**	*Hev b 12* (Latex rubber)<br>*Mal d 3* (Apple)<br>*Mus a 3* (Banana)<br>*Pru p 3* (Peach)<br>*Sola l 3* (Tomato)<br>*Tri a 14* (Wheat)	Food<br>Food<br>Food<br>Food<br>Food
**A0A7J6H3P5**	**Aldehyde dehydrogenase**	2.99E+09	1.39E+09	2.56E+09	**5.40E+09**	*Alt a 10* (Fungi-Ascomycete)<br>*Cla h 10* (Fungi-Ascomycete)<br>*Har a 2* (Ladybeetle)<br>*Tyr p 35* (Storage mite)	Airway<br>Airway<br>Airway<br>Airway
**A0A7J6GTH1<br>A0A7J6I6F2<br>A0A7J6FAG8<br>A0A7J6DL32**		1.29E+07	*N.D.*	*N.D.*	**1.09E+08**		
**A0A7J6FQ30<br>A0A7J6DLS9<br>A0A7J6HHA5**		*N.D.*	*N.D.*	*N.D.*	**5.38E+07**		
**A0A7J6GYE1**	**Ferritin**	2.03E+09	1.38E+09	1.55E+09	**2.94E+09**	*Der f 30* (House dust mite)<br>*Der p 30* (House dust mite)	Airway<br>Airway
**A0A7J6H5H4**	**SERPIN domain-containing protein**	0.69E+09	**1.31E+09**	0.93E+09	1.04E+09	*Der f 27* (House dust mite)<br>*Tri a 33* (Wheat)	Airway<br>Airway

**A0A7J6EKG1 A0A7J6ENE6 A0A7J6EKM6**	**(1->3)-beta-glucan endohydrolase**	0.38E+10	0.13E+10	**1.12E+10**	1.15E+09	*Hev b 2* (Latex rubber) *Mus a 5* (Banana)	Contact Food
**A0A7J6EAU5**		1.79E+09	0.94E+09	1.82E+09	**2.51E+09**		
**A0A7J6E4K5**		1.89E+08	**6.98E+08**	5.97E+08	2.00E+08		
**A0A7J6EUT7**		2.16E+08	**5.50E+08**	4.00E+08	1.44E+08		
**A0A7J6EAV2 A0A7J6EF61 A0A7J6E8S1**		0.57E+09	0.53E+09	**3.08E+09**	4.66E+08		
**A0A7J6E8M2 A0A7J6ED18**		*N.D.*	**4.94E+08**	0.04E+08	9.19E+06		
**A0A7J6DMI6**		2.66E+08	4.45E+08	**4.85E+08**	2.69E+08		
**A0A7J6IAD9**		2.47E+08	**3.76E+08**	3.16E+08	3.02E+08		
**A0A7J6EGQ4 A0A7J6EY25**		0.57E+08	1.67E+08	0.83E+08	*N.D.*		
**A0A7J6GT01**		0.43E+08	1.47E+08	**1.60E+08**	1.40E+08		
**A0A7J6DP61**		0.72E+08	**1.15E+08**	0.96E+08	4.50E+07		
**A0A7J6HQX0**		*N.D.*	1.14E+08	0.65E+08	**1.57E+08**		
**A0A7J6HJN1**		3.02E+07	7.18E+07	9.76E+07	**1.05E+08**		
**A0A7J6H4Q0**		2.80E+07	**7.07E+07**	4.06E+07	1.56E+07		
**A0A7J6FD71**		7.54E+07	6.71E+07	**9.47E+07**	*N.D.*		

**A0A7J6DX44**		4.70E+07	6.42E+07	5.60E+07	**1.44E+08**		
**A0A7J6HXP0**		4.35E+07	5.03E+07	4.47E+07	**4.34E+08**		
**A0A7J6HX51 A0A7J6GXU4**		8.61E+07	5.02E+07	**9.81E+07**	7.07E+07		
**A0A7J6GB53 A0A7J6FB88**		1.97E+07	3.58E+07	2.61E+07	**2.53E+08**		
**A0A7J6FI62 A0A7J6F9Z7**		2.29E+07	2.31E+07	3.53E+07	**1.09E+08**		
**A0A7J6GWF6**		*N.D.*	**1.96E+07**	*N.D.*	*N.D.*		
**A0A7J6H3Z8**		0.50E+07	1.01E+07	**4.96E+07**	*N.D.*		
**A0A7J6FJI4 A0A7J6DR16 A0A7J6DR07**		8.05E+06	*N.D.*	9.56E+06	**4.71E+07**		
**A0A7J6FD71 A0A7J6HX99 A0A7J6G837**		*N.D.*	*N.D.*	*N.D.*	**6.61E+07**		
**A0A7J6FXV3 A0A7J6EFE3**		*N.D.*	*N.D.*	*N.D.*	**1.30E+07**		
**A0A7J6HXW1**		*N.D.*	*N.D.*	*N.D.*	**1.25E+07**		
**A0A7J6HB83**	**Calreticulin**	0.71E+09	1.06E+09	1.55E+09	**5.09E+09**	*Pen ch 31* (Fungi-Ascomycete)	Airway
**A0A7J6HB40 A0A7J6ELP6**	**Superoxide dismutase [Cu-Zn]**	2.03E+09	0.99E+09	**2.59E+09**	1.08E+09	*Ole e 5* (Olive)	Airway
**A0A7J6G1D4**		*N.D.*	*N.D.*	*N.D.*	**1.79E+09**		

**A0A7J6FFH3**		*N.D.*	*N.D.*	*N.D.*	**1.64E+07**		
**A0A7J6H7M3**	**Transaldolase**	9.54E+08	8.70E+08	8.82E+08	**1.93E+09**	*Cla c 14* (Fungi-Ascomycete)<br>*Fus p 4* (Fungi-Ascomycete)<br>*Pen ch 35* (Fungi-Ascomycete)	Airway<br>Airway<br>Airway
**A0A7J6IAQ6**		2.42E+08	**4.43E+08**	2.20E+08	3.43E+08		
**A0A7J6GLE4 A0A7J6FKP2 A0A7J6FA10**	**Peptidyl-prolyl isomerase**	2.30E+08	**8.23E+08**	5.99E+08	2.42E+08	*Asp f 11* (Fungi-Ascomycete)	Airway
**A0A7J6FHX4 A0A7J6GJU3 A0A7J6E5K1**		2.02E+08	1.84E+08	2.70E+08	**7.40E+08**		
**A0A7J6GB79**		**1.72E+08**	1.50E+08	0.93E+07	*N.D.*		
**A0A7J6FRD7**		0.90E+08	1.08E+08	**1.35E+08**	1.22E+07		
**A0A7J6G5Y5**		1.12E+08	1.06E+08	1.32E+08	**2.76E+08**		
**A0A7J6E8B7**		6.10E+07	**9.19E+07**	4.23E+07	2.09E+07		
**A0A7J6FHQ5 A0A7J6DRN2**		6.05E+07	3.72E+07	5.18E+07	**2.02E+08**		

**A0A7J6HL15 A0A7J6GBJ4**		**5.58E+07**	3.63E+07	3.47E+07	3.08E+07		
**A0A7J6FIT5**		*N.D.*	**3.57E+07**	*N.D.*	*N.D.*		
**A0A7J6E7Z6 A0A7J6E0U6**		4.43E+07	2.73E+07	2.59E+07	**9.76E+07**		
**A0A7J6I417**		5.20E+06	5.51E+06	*N.D.*	**1.27E+07**		
**A0A7J6FIE1**		7.30E+06	*N.D.*	**7.63E+06**	*N.D.*		
**A0A7J6H5W6**		*N.D.*	*N.D.*	*N.D.*	**9.82E+08**		
**A0A7J6GIP5**	**Chitin-binding type-1 domain-containing protein**	4.85E+09	3.62E+09	**1.05E+10**	2.81E+09	*Der f 18* (House dust mite)<br><br>*Der p 18* (House dust mite)	Airway<br><br>Airway
**A0A7J6I6L6**		0.59E+09	0.70E+09	**1.01E+09**	5.65E+08		
**A0A7J6HSM0**		2.66E+08	2.03E+08	3.87E+08	**1.06E+09**		
**A0A7J6G347**		2.56E+08	0.51E+08	**4.75E+08**	4.52E+08		
**A0A7J6I847**		5.47E+07	4.86E+07	**6.48E+07**	*N.D.*		
**A0A7J6H5Q7 A0A7J6IBH0**		*N.D.*	0.84E+07	9.16E+07	**1.30E+09**		

**A0A7J6IBR9 A0A7J6IC31 A0A7J6IAL0**		8.02E+07	0.76E+07	4.79E+07	**1.30E+09**		
**A0A7J6EH05**		*N.D.*	*N.D.*	**3.72E+07**	*N.D.*		
**A0A7J6FLX5**		*N.D.*	*N.D.*	**3.51E+08**	*N.D.*		
**A0A7J6EFZ4**		*N.D.*	*N.D.*	*N.D.*	**1.50E+07**		
**A0A7J6H1D6**	**L-ascorbate peroxidase**	0.73E+08	**6.28E+08**	3.17E+08	3.86E+07	*Mus a 6* (Banana)	Food
**A0A7J6HD14**		4.53E+09	8.65E+09	8.72E+09	**1.04E+10**		
**A0A7J6GTH6**		7.58E+08	3.34E+08	7.34E+08	**8.42E+08**		
**A0A7J6G6C0 A0A7J6F1W2**		4.98E+08	2.60E+08	2.70E+08	**1.64E+09**		
**A0A7J6FIL3**		0.81E+08	0.81E+08	**1.90E+08**	*N.D.*		
**A0A7J6HAM9**		*N.D.*	*N.D.*	*N.D.*	**1.31E+08**		
**A0A7J6HW67**	**Cystatin-domain containing protein**	3.55E+08	5.29E+08	**5.65E+08**	2.66E+08	*Can f 8* (Dog) *Fel d 3* (Cat)	Airway Airway
**A0A7J6FUY4 A0A7J6EHC3**		*N.D.*	*N.D.*	*N.D.*	**4.48E+07**		
**A0A7J6I1W7**	**Cytochrome c-domain containing protein**	4.22E+08	5.27E+08	4.95E+08	**1.71E+09**	*Cur l 3* (Fungi-Ascomycete)	Airway

**A0A7J6I2T9**		2.00E+08	3.29E+08	3.72E+08	**6.63E+08**		
**A0A7J6I6Q5**	**Pyruvate kinase**	6.48E+08	4.36E+08	8.12E+08	**1.46E+09**	*Pan h 9* (Striped catfish)	Food
**A0A7J6ERQ0**		5.15E+08	3.52E+08	7.38E+08	**8.51E+08**		
**A0A7J6DYV9 A0A7J6H2G3 A0A7J6GGC3**		2.29E+07	3.75E+07	1.81E+07	**3.76E+07**		
**A0A7J6I003**		3.65E+07	1.97E+07	1.30E+07	**1.00E+09**		
**A0A7J6FR44 A0A7J6G7G2**		1.06E+08	1.27E+07	4.97E+07	**2.04E+08**		
**A0A7J6FBC2**		6.56E+07	*N.D.*	2.03E+07	*N.D.*		
**A0A7J6I9Q3**		*N.D.*	*N.D.*	*N.D.*	**1.59E+07**		
**A0A7J6I9W7**	**Glucose-6-phosphate isomerase**	2.86E+08	3.89E+08	**5.50E+08**	3.37E+08	*Pan h 11* (Striped catfish)	Food
**A0A7J6EVW4**		3.15E+08	1.64E+08	1.74E+08	**4.77E+08**		
**A0A7J6HKD1**	**Eukaryotic translation initiation factor 3**	2.00E+08	**3.83E+08**	2.14E+08	1.77E+08	*For t 2* (Biting midge)	Systemic

**A0A7J6G256 A0A7J6HPQ8 A0A7J6ESZ1 A0A7J6I4V0**		3.16E+08	2.81E+08	2.13E+08	**6.36E+08**		
**A0A7J6GJB4**		0.73E+08	**1.44E+08**	0.91E+08	8.98E+07		
**A0A7J6EJL9 A0A7J6GJ62 A0A7J6IDC4**		1.90E+08	1.44E+08	2.15E+08	8.28E+08		
**A0A7J6EAF0**		1.70E+08	1.18E+08	1.72E+08	**7.66E+08**		
**A0A7J6GNI4**		1.45E+08	0.77E+08	0.44E+08	**1.69E+08**		
**A0A7J6FZF8 A0A7J6HQU3**		**8.83E+07**	7.65E+07	5.99E+07	4.87E+08		
**A0A7J6GU50 A0A7J6GMZ3**		9.48E+07	5.66E+07	5.58E+07	**2.04E+08**		
**A0A7J6GXB8 A0A7J6E2F6 A0A7J6I306**		1.37E+08	0.33E+08	0.82E+08	**2.75E+08**		
**A0A7J6GY94 A0A7J6EZI0**		**6.71E+07**	2.30E+07	5.70E+07	1.91E+07		
**A0A7J6DSH8 A0A7J6ECG4**		3.21E+07	*N.D.*	2.41E+07	**3.10E+08**		

**A0A7J6HEP2**		4.54E+07	*N.D.*	3.25E+07	**4.29E+08**		
**A0A7J6IBH6**		5.50E+07	*N.D.*	2.96E+07	**3.39E+08**		
**A0A7J6GR38**	**Beta-fructofuranosidase**	0.76E+07	**3.68E+08**	2.75E+08	1.08E+08	*Sola l 12* (Tomato)	Food
**A0A7J6DSF0**	**Cysteine protease inhibitor**	7.00E+08	3.03E+08	2.65E+08	**1.52E+09**	*Ani s 4* (Herring worm)<br>*Sola t 3* (Potato)	Food<br>Food
**A0A7J6G6H4**<br>**A0A7J6ENT1**<br>**A0A7J6F296**		2.24E+08	2.58E+08	1.32E+08	**4.89E+08**		
**A0A7J6FLP0**	**Pectate lyase**	1.03E+08	**2.80E+08**	1.18E+08	1.26E+08	*Amb a 1* (Short ragweed)<br>*Art v 6* (Wormwood)<br>*Cry j 1* (Japanese cedar)<br>*Cup s 1* (Common cypress)<br>*Hel a 6* (Sunflower)	Airway<br>Airway<br>Airway<br>Airway<br>Airway
**A0A7J6HTG5**		*N.D.*	**1.78E+07**	*N.D.*	1.11E+07		

**A0A7J6F9E2**		1.45E+07	1.59E+07	*N.D.*	**1.08E+08**	*Jun a 1* (Mountain cedar)<br><br>*Pen c 32* (Fungi-Ascomycete)	Airway<br><br>Airway
**A0A7J6I2E5**		*N.D.*	*N.D.*	*N.D.*	**1.16E+08**		
**A0A7J6GVP7**<br>**A0A7J6G545**		*N.D.*	*N.D.*	*N.D.*	**4.98E+06**		
**A0A7J6H491**	**Profilin**	2.45E+08	2.05E+08	2.08E+08	**5.80E+08**	*Amb a 8* (Short ragweed)<br><br>*Ara h 5* (Peanut)<br><br>*Bet v 2* (White birch)<br><br>*Cor a 2* (Hazelnut)	Airway<br><br>Food<br><br>Airway<br><br>Food

**A0A7J6EUM0<br>A0A7J6HTU8**		*N.D.*	*N.D.*	*N.D.*	**3.50E+07**	*Cyn d 12* (Bermuda grass)<br>*Gly m 3* (Soybean)<br>*Hev b 8* (Short ragweed)<br>*Mal d 4* (Apple)<br>*Pru p 4* (Peach)<br>*Sola l 1* (Tomato)	Airway<br>Food<br>Contact<br>Food<br>Food<br>Food
**A0A7J6FVM7**	**Tubulin alpha chain**	**1.51E+09**	1.03E+09	1.08E+09	2.64E+08	*Der f 33* (House dust mite)<br>*Der p 33* (House dust mite)	Airway<br>Airway
**A0A7J6EY26**		**1.25E+09**	0.20E+09	0.23E+09	2.65E+08		
**A0A7J6H710**		4.32E+07	2.92E+07	5.20E+07	**1.47E+09**		
**A0A7J6GA12<br>A0A7J6FJQ0**	**Alpha-amylase**	*N.D.*	**1.63E+08**	0.15E+08	*N.D.*	*Bla g 11* (Cockroach)<br>*Blo t 4* (Storage mite)<br>*Der f 4* (House dust mite)	Airway<br>Airway<br>Airway

						Der p 4 (House dust mite)<br><br>*Hor v 16* (Barley)<br><br>*Per a 11* (Cockroach)	Airway<br><br>Food<br><br>Airway
**A0A7J6F8W0**	**Expansin**	0.77E+08	**1.54E+08**	0.75E+08	1.39E+08	*Cyn d 1* (Bermuda grass)<br><br>*Lol p 1* (Rye grass)<br><br>*Ory s 1* (Rice)<br><br>*Phl p 1* (Timothy grass)<br><br>*Poa p 1* (Kentucky blue grass)<br><br>*Zea m 1* (Maize)	Airway<br><br>Airway<br><br>Airway<br><br>Airway<br><br>Airway<br><br>Airway
**A0A7J6GYI3**<br>**A0A7J6GCT4**		0.75E+08	**1.57E+08**	1.15E+08	3.09E+07		
**A0A7J6HHP6**<br>**A0A7J6I6H0**		**1.00E+08**	0.95E+08	**1.00E+08**	5.06E+07		
**A0A7J6DLM1**		*N.D.*	7.50E+07	**8.72E+07**	*N.D.*		
**A0A7J6GS30**		*N.D.*	**3.45E+07**	1.02E+07	*N.D.*		
**A0A7J6HHF7**		*N.D.*	2.89E+07	*N.D.*	**2.92E+07**		
**A0A7J6GND6**<br>**A0A7J6FBP3**		1.06E+07	1.56E+07	*N.D.*	**1.52E+08**		
**A0A7J6FWX7**		2.58E+07	*N.D.*	**4.55E+07**	*N.D.*		
**A0A7J6H0I3**		*N.D.*	*N.D.*	4.85E+07	7.71E+06		

**A0A7J6HGI1**	**Beta-amylase**	0.03E+08	**1.26E+08**	1.07E+08	*N.D.*	*Hor v 17* (Barley)<br>*Tri a 17* (Wheat)	Food<br>Food
**A0A7J6F9B7 A0A7J6FJV6**		*N.D.*	*N.D.*	*N.D.*	**1.32E+07**		
**A0A7J6GWH8**		*N.D.*	*N.D.*	*N.D.*	**1.32E+07**		
**A0A7J6HG73**	**Bet v 1-domain containing protein (Pathogenesis-related protein, PR-10)**	1.23E+10	1.08E+10	**2.86E+10**	2.46E+10	*Ara h 8* (Peanut)<br>*Bet v 1* (White birch)<br>*Cor a 1* (Hazelnut)<br>*Gly m 4* (Soybean)<br>*Mal d 1* (Apple)<br>*Pru p 1* (Peach)<br>*Sola l 4* (Tomato)	Food<br>Airway<br>Airway<br>Food<br>Food<br>Food<br>Food
**A0A7J6F1J6**		1.70E+08	1.10E+08	**1.82E+08**	7.37E+07		
**A0A7J6EBP3**		*N.D.*	2.89E+07	2.42E+07	**4.20E+07**		
**A0A7J6F1K3**		8.94E+07	*N.D.*	*N.D.*	**3.06E+08**		
**A0A7J6HLD9**		*N.D.*	*N.D.*	*N.D.*	**2.18E+10**		

**A0A7J6HEQ6**		*N.D.*	*N.D.*	*N.D.*	**2.73E+08**		
**A0A7J6HG14**		*N.D.*	*N.D.*	*N.D.*	**4.69E+07**		
**A0A7J6HDD3**<br>**A0A7J6HFA3**		*N.D.*	*N.D.*	*N.D.*	**3.79E+07**		
**A0A7J6I6E2**		*N.D.*	*N.D.*	*N.D.*	**8.10E+06**		
**A0A7J6FGV0**	**Phospholipase A1**	**4.03E+08**	0.82E+08	*N.D.*	3.07E+08	*Pol e 1* (Wasp)<br>*Vesp v 1* (Asian hornet)<br>*Ves m 1* (Yellow jacket)	Systemic<br>Systemic<br>Systemic
**A0A7J6IAY5**		**4.03E+08**	0.82E+08	*N.D.*	1.40E+07		
**A0A7J6H0B6**		**4.03E+08**	0.82E+08	*N.D.*	1.19E+07		
**A0A7J6HXF0**		0.62E+07	1.08E+07	**1.24E+07**	4.49E+06		

**A0A7J6G3Y7 A0A7J6F236 A0A7J6EPM4**		**4.53E+07**	*N.D.*	*N.D.*	*N.D.*		
**A0A7J6H2M1**	**Lactate dehydrogenase**	0.53E+07	3.46E+07	5.11E+07	**1.35E+08**	*Pan h 10* (Striped catfish)	Food
**A0A7J6GLR3 A0A7J6FBQ5 A0A7J6GLQ5 A0A7J6H4N6 A0A7J6GLT4 A0A7J6E325 A0A7J6FEC2**	**(R)-mandelonitrile lyase**	3.49E+08	0.31E+08	**6.63E+08**	4.40E+07	*Pru du 10* (Almond)	Food
**A0A7J6GC68 A0A7J6FYG8**		1.05E+08	0.23E+08	1.00E+08	**4.52E+08**		
**A0A7J6EMQ7**		**2.12E+07**	1.78E+07	*N.D.*	*N.D.*		
**A0A7J6HN73 A0A7J6H0Z2**		**4.44E+07**	*N.D.*	2.47E+07	2.25E+07		
**A0A7J6GLW1**	**GMC_Oxidoredutase_N domain-containing protein**	**2.12E+07**	1.78E+07	*N.D.*	*N.D.*	*Mala s 12* (Skin yeast)	Contact

**A0A7J6FK77**	**ML domain-containing protein**	*N.D.*	1.29E+07	**1.68E+07**	*N.D.*	*Blo t 2* (Storage mite)	Airway
**A0A7J6E1L6 A0A7J6E5W1**	**Endo-1,3(4)-beta-glucanase**	*N.D.*	*N.D.*	**1.13E+07**	*N.D.*	*Hev b 2* (Latex rubber)<br>*Mus a 5* (Banana)	Contact<br>Food
**A0A7J6F7K7 A0A7J6GPK7 A0A7J6H2Q1 A0A7J6GR85**		*N.D.*	*N.D.*	*N.D.*	**6.04E+07**		
**A0A7J6I550**		*N.D.*	*N.D.*	*N.D.*	**1.86E+07**		
**A0A7J6I9Z8**		*N.D.*	*N.D.*	*N.D.*	**1.54E+07**		
**A0A7J6H1U8**	**Agglutinin domain-containing protein**	1.82E+09	4.07E+08	6.96E+08	**2.54E+09**	*Tri a 18* (Wheat)	Food
**A0A7J6G4B7 A0A7J6F1V1**		**7.09E+07**	*N.D.*	0.72E+07	3.35E+07		
**A0A7J6FGU9**		*N.D.*	*N.D.*	1.39E+08	**2.54E+09**		

**A0A7J6FBU3**	**X8 domain-containing protein**	5.21E+06	*N.D.*	*N.D.*	**1.00E+08**	*Ole e 10* (Olive)	Airway
**A0A7J6HRL7**	**Non-specific serine/threonine protein kinase**	1.06E+07	*N.D.*	*N.D.*	**5.95E+07**	*For t 1* (Biting midge)	Systemic
**A0A7J6GSM4**		*N.D.*	*N.D.*	*N.D.*	**5.29E+07**		
**A0A7J6DSK9**		*N.D.*	*N.D.*	*N.D.*	**4.93E+07**		
**A0A7J6ELT8<br>A0A7J6GN21<br>A0A7J6FJW2**		*N.D.*	*N.D.*	*N.D.*	**1.05E+07**		
**A0A7J6FL68<br>A0A7J6F113<br>A0A7J6FAW1<br>A0A7J6E405<br>A0A7J6FUU9<br>A0A7J6HGC3**		*N.D.*	*N.D.*	*N.D.*	**4.66E+06**		
**A0A7J6H6B1**		*N.D.*	*N.D.*	*N.D.*	**1.10E+08**		

**A0A7J6FRH4<br>A0A7J6EPV2**	**EF-hand domain-containing protein**	0.80E+08	0.53E+08	1.25E+08	**2.16E+08**	*Cra c 4* (North Sea shrimp)<br>*Cra a 4* (Pacific oyster)<br>*Pen m 4* (Black Tiger shrimp)<br>*Pon l 4* (Crayfish)<br>*Scy p 4* (Mud crab)	Food<br>Food<br>Food<br>Food<br>Food
**A0A7J6I7N3**		3.22E+06	*N.D.*	*N.D.*	**1.52E+07**		
**A0A7J6EYA1<br>A0A7J6GQN3<br>A0A7J6GED9<br>A0A7J6EJR9**		*N.D.*	*N.D.*	*N.D.*	**2.39E+07**		
**A0A7J6GJR5**	**Carboxypeptidase**	0.39E+09	**1.43E+09**	0.93E+09	6.89E+08	*Api m 9* (Honeybee)	Systemic
**A0A7J6I4U3**		2.23E+08	**1.07E+09**	7.85E+08	2.94E+08		
**A0A7J6FRW9**		1.03E+08	**3.38E+08**	2.73E+08	4.06E+07		
**A0A7J6HYY7**		3.27E+08	3.09E+08	3.57E+08	**7.59E+08**		
**A0A7J6HQS1<br>A0A7J6GS12**		1.30E+08	1.43E+08	0.63E+08	**1.92E+08**		

**A0A7J6E757 A0A7J6GXF1**		0.55E+08	1.26E+08	**1.29E+08**	5.39E+07		
**A0A7J6EJT5 A0A7J6EVP8**		1.39E+07	7.26E+07	*N.D.*	**1.28E+08**		
**A0A7J6H6U1**		*N.D.*	**4.90E+07**	2.24E+07	*N.D.*		
**A0A7J6H7L2**		*N.D.*	**4.72E+07**	*N.D.*	*N.D.*		
**A0A7J6E0D4**		0.61E+07	**3.07E+07**	1.42E+07	8.30E+06		
**A0A7J6H4X1**		*N.D.*	**1.35E+07**	0.81E+07	*N.D.*		
**A0A7J6GH52 A0A7J6EU30**		*N.D.*	**1.14E+07**	*N.D.*	*N.D.*		
